# Complex formation of fenchone with α-cyclodextrin: NMR titrations

**DOI:** 10.1007/s10847-013-0356-4

**Published:** 2013-08-10

**Authors:** Michał Nowakowski, Andrzej Ejchart

**Affiliations:** 1 Faculty of Chemistry, Warsaw University, Pasteura 1, 02-093 Warsaw, Poland; 2 Institute of Biochemistry and Biophysics, Polish Academy of Sciences, Pawińskiego 5A, 02-106 Warsaw, Poland

**Keywords:** Alpha-cyclodextrin, Fenchone, Inclusion complexes, ^13^C NMR titration, Sequential association constants, Diastereomeric complexes, Chiral recognition

## Abstract

^13^C NMR titration studies of inclusion complexes of bicyclic terpenoid, fenchone enantiomers with α-cyclodextrin revealed their 1:2 guest–host stoichiometry. Sequential binding constants were determined indicating a strong binding cooperativity of two α-cyclodextrin to fenchone. The overall association constants were used to calculate the Gibbs free energies of diastereomeric complex formation, which might be used as a measure of chiral recognition of fenchone by α-cyclodextrin. These results were compared with corresponding data derived for camphor, which is an isomeric bicyclic terpenoid.

**Electronic supplementary material:**

The online version of this article (doi:10.1007/s10847-013-0356-4) contains supplementary material, which is available to authorized users.

## Introduction

Cyclodextrins (CDs) are macrocyclic oligosaccharides composed of a number of glucopyranoside units bound together by α-1,4 bonds. The naturally occurring α-, β- and γ-cyclodextrins (αCD, βCD, and γCD) consist of six, seven, and eight glucopyranose units, respectively [1]. They are obtained by enzymatic starch degradation [1, 2]. CDs, whose shape remains a truncated cone, contain a lipophilic central cavity and a hydrophilic outer surface. The size of αCD cavity: bottom diameter 0.53 nm, top diameter 0.47 nm, and cone height 0.79 nm [1, 2] allows for accommodating many low molecular weight compounds. In aqueous solutions, CDs can form host–guest inclusion complexes with many partially or fully lipophilic molecules often increasing the guest solubility. Hence their wide application in chemistry, pharmacy, or food industry [1–3]. A number of non-covalent forces is responsible for the stabilization of inclusion complexes [4]. The stoichiometry and stability of such complexes strongly depend on the physicochemical properties of guest molecules [5].

**Fig. 1:**
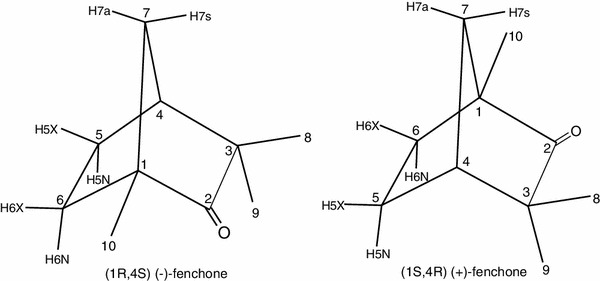
Enantiomers of fenchone

Among many compounds complexed with CDs and their derivatives, the bicyclic monoterpenoid, camphor, has been extensively studied by different experimental methods [6–17]. In contrast CD complexes of camphor isomer - fenchone (1,3,3-trimethylbicyclo[2.2.1]heptan-2-one) have been the subject of few studies mainly devoted to physiological or pharmaceutical applications [18–23]. Hence rigorous physicochemical studies of fenchone—CDs complexes would provide useful information on the variation of complex stabilities with variation in the geometry of isomeric guest compounds. Fenchone is characterized by low solubility in water and a size that is comparable to that of the inner cavity of αCD. Fenchone enantiomers are shown in Fig. 1.

NMR spectroscopy is very well suited to study weak and moderate strength molecular complexes and their properties. It is accepted that taking into account typical NMR sample concentrations, the best accuracy can be obtained for association constants within the range 10–10^6^ M^−1^ [24, 25]. Therefore, NMR has been widely used for studying inclusion complexes formed by CDs. The success of NMR spectroscopy in this field is due to its ability to study complex chemical systems, to determine complex stoichiometry, association constants, and conformations and to obtain information on their symmetry and dynamics [5, 26, 27]. Compared to other techniques, NMR spectroscopy provides a superior method to study complexation phenomena as guest and host molecules can be simultaneously observed at the atomic level. Since the rates of complex formation and decomposition are usually faster than the chemical shift time scale (often misleadingly named NMR time scale), the observed chemical shifts are the mole fraction weighted averages of the chemical shifts existing in the free and complexed molecules [5, 24]. If the assumption of rapid equilibrium is not valid, an analysis of the total lineshape is required [16, 28]. CDs are chiral and, therefore, can form diastereomeric complexes, usually of different stability, with enantiomeric species [29].

## Experimental

### NMR measurements

αCD (Sigma, 99 % purity) and both enantiomers of fenchone (the gift from prof. H. Dodziuk) were used without further purification. The ^2^H_2_O (Armar Chemicals, 99.8 at.% D) solutions of fenchone enantiomers contained small amount of acetone (Chempur, pure p.a.) whose NMR signal was used as the indicator of external magnetic field inhomogeneity and internal secondary reference: δ_H_ = 2.22 and δ_C_ = 30.89 [30]. All measurements were performed at magnetic field of 9.4 T, using a Varian Unity Inova 400 MHz, spectrometer. NMR measurements were performed at a temperature carefully adjusted to 300.6 K with an accuracy of 0.1 K and was checked by an ethylene glycol reference sample (composition: 80 % ethylene glycol, Aldrich/20 % dimethyl sulfoxide-D_6_, Armar Chemicals).

### Titration of fenchone enantiomers with αCD

(+)- and (−)-fenchone were dissolved in D_2_O to a concentration of 1 mM. Part of each solution was separated from the rest and αCD was added in large excess over fenchone. For each fenchone enantiomer these basic solutions were mixed afterwards together in order to prepare NMR samples of various αCD/fenchone molar ratios so that the concentrations of fenchone enantiomers remained constant during the titrations. Accurate values of molar ratios were as follows: {2.66, 5.81, 10.35, 16.46, 22.41, 27.30, 39.39, 61.75} for αCD/(+)-fenchone and {3.63, 7.79, 16.46, 25.13, 33.86, 40.54, 50.48, 67.95, 81.65} for αCD/(−)-fenchone. These values were checked by signal integration of six anomeric protons of αCD versus nine methyl protons of fenchone. Both, 1D ^1^H and 2D ^1^H/^13^C HSQC spectra were recorded for each solution. 2D ^1^H/^13^C HSQC spectra were measured with sweep width of indirect f_1_ dimension equal to 25 ppm in order to achieve high digital resolution of ^13^C dimension in feasible measurement time. Examples of 1D ^1^H NMR spectra of αCD/(−)-fenchone (molar ratio 3.63) and αCD/(+)-fenchone (molar ratio 2.66) mixtures are given in Figs. SF1 and SF2, respectively (Supplementary Materials).

### Determination of association constants

The changes in ^1^H and ^13^C chemical shifts of three methyl signals as a function of αCD concentration were analyzed assuming either simple 1:1 or complex 1:1 and 1:2 guest–host stoichiometry. In the latter case stepwise (sequential) binding [26] was assumed. Sequential macroscopic association constants were defined by the following eqns.:

$$ K_{1,c} = {{\left[ {\text{GH}} \right]} \mathord{\left/ {\vphantom {{\left[ {\text{GH}} \right]} {\left[ {\text{G}} \right]\left[ {\text{H}} \right]}}} \right. \kern-0pt} {\left[ {\text{G}} \right]\left[ {\text{H}} \right]}} ,\quad K_{2,c} = {{\left[ {{\text{GH}}_{2} } \right]} \mathord{\left/ {\vphantom {{\left[ {{\text{GH}}_{2} } \right]} {\left[ {\text{GH}} \right]\left[ {\text{H}} \right]}}} \right. \kern-0pt} {\left[ {\text{GH}} \right]\left[ {\text{H}} \right]}} $$

with square brackets [.] denoting molar concentrations of appropriate species, [G]—guest (fenchone), [H]—host (αCD), [GH] and [GH_2_]—complexes with stoichiometry 1:1 and 1:2, respectively. Averaged chemical shifts, *δ*_*a*_, were calculated using the formula [24]

$$ \delta_{a} = \delta_{f} + \sum\limits_{i = 1}^{N} {x_{i} (\delta_{i} - \delta_{f} )} = \delta_{f} + \sum\limits_{i = 1}^{N} {x_{i} \Updelta \delta_{i} } $$

where *δ*_*f*_ is chemical shift in uncomplexed fenchone, whereas *x*_*i*_ and *δ*_*i*_ are mole fractions and chemical shifts of *i*-th complex species. Association constants *K*_*i,c*_ and complexation-induced shifts Δ*δ*_*i*_ were determined by fitting the experimental dependence of *δ*_*exp*_ in fenchone molecules versus *M* various concentrations of αCD. The least-squares procedure used a Fortran routine written in-house optimizing the model parameters that consisted of minimization through a grid search of the target function χ^2^ given by:

$$ \chi^{2} = \sum\limits_{i = 1}^{M} {(\delta_{\exp } - \delta_{a} )^{2} } $$

Confidence limits of fitted parameters were estimated by use of constant χ^2^ boundaries [31]. Fisher–Snedecor statistics (*F* test) was used for the stoichiometry selection at the probability 0.01.

## Results and discussion

**Fig. 2:**
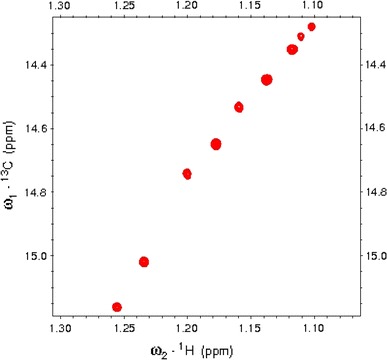
Superposition of a series of ^1^H/^13^C HSQC spectra showing C10 methyl correlations in (−)fenchone–αCD complex. The αCD/(−)fenchone molar ratio was changed from 0 (*upper right side*) to 81.7 (*lower left side*). The observed Δδ_C_ and Δδ_H_ were equal to 0.896 and 0.149 ppm, respectively

The fenchone signal assignments had to be done *de novo* on the basis of COSY, NOESY and ^1^H/^13^C HSQC spectra since the literature values [32, 33] corresponded to a different solvent. ^1^H and ^13^C chemical shifts of free fenchone are collected in Table 1. 1D ¹H-NMR and 2D ^1^H/^13^C HSQC spectra for (−)-fenchone in D_2_O are shown in Figs SF3 and SF4, respectively (Supplementary Materials). Three methyl signals exhibit by far the largest and easiest to detect ^1^H and ^13^C chemical shift changes on complexation. Their ^13^C resonances with complexation shifts, exceeding those of ^1^H signals, are especially convenient for quantitative analysis of NMR titration data. In order to provide satisfactory signal dispersion and signal-to-noise ratios of fenchone methyls at the concentration of 1 mM and the natural abundance of ^13^C isotope, the 2D ^1^H/^13^C correlation spectra with ^1^H detection are the method of choice. Superposition of a series of HSQC spectra showing C10 correlations in (−)fenchone–αCD complex is shown in Fig. 2. So derived ^13^C methyl chemical shift changes upon variable ratios of αCD to fenchone enantiomers were used in a numerical procedure yielding best estimates of the association constants.

**Table 1:** ^1^H and ^13^C chemical shifts of fenchone in D_2_O and CDCl_3_ solutions

Position	^1^H^a^	^1^H^b^	^1^H^c^	^13^C^a^	^13^C^b^
4	2.19	2.14	2.14	45.84	45.3
5x	1.76	1.72	1.80	24.71	25.0
5n	1.76	1.82	1.70		
6x	1.68	1.56	1.54	32.41	31.8
6n	1.34	1.39	1.37		
7a	1.61	1.53	1.54	41.81	41.6
7s	1.89	1.79	1.80		
8	1.04	1.03	1.04	22.89	23.3
9	1.03	1.03	1.04	21.37	21.7
10	1.11	1.14	1.15	14.16	14.6

^a^D_2_O solution, this work

^b^CDCl_3_ solution, Ref. [32]

^c^CDCl_3_ solution. Ref [33]

**Fig. 3:**
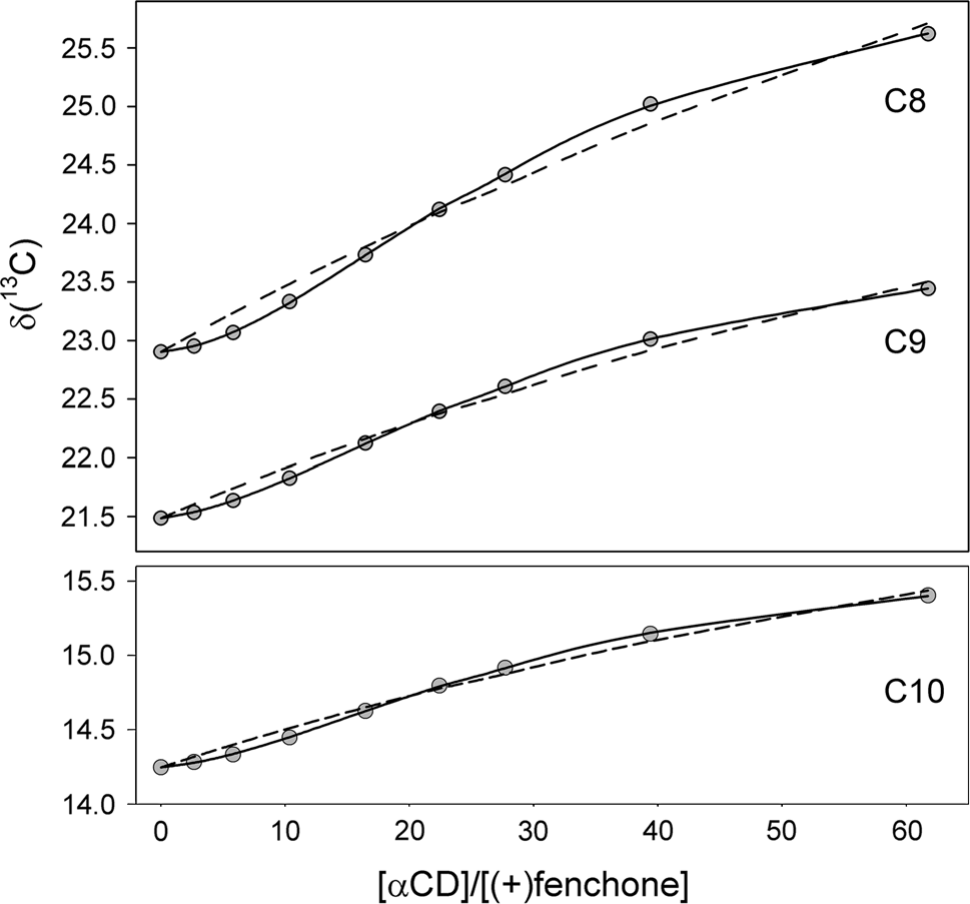
Experimental ^13^C chemical shifts (*gray circles*) measured for methyl carbons of (+)fenchone in NMR titration with αCD. *Solid lines* represent the best fit curves for the stepwise binding (complex stoichiometries 1:1 and 1:2). *Dashed lines* correspond to 1:1 complex stoichiometry

**Fig. 4:**
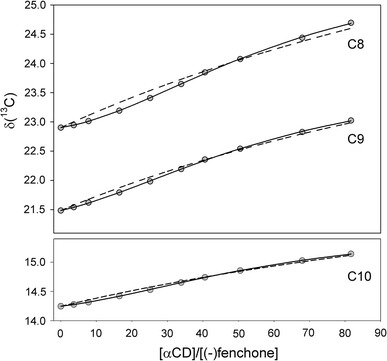
Experimental ^13^C chemical shifts (*gray circles*) measured for methyl carbons of (−)fenchone in NMR titration with αCD. *Solid lines* represent the best fit curves for the stepwise binding (complex stoichiometries 1:1 and 1:2). *Dashed lines* correspond to 1:1 complex stoichiometry

The sigmoidal shape of all titration curves (Figs. 3, 4) strongly suggests a composite stoichiometry of the studied complexes and a possibility of cooperative binding [34, 35]. In fact, the best reproduction of experimental chemical shifts has been obtained assuming a sequential binding model, whereas a simple 1:1 stoichiometry was precluded on the basis of Fisher–Snedecor statistics. The best fit estimates of the association constants are collected in Table 2. Their *K*_1,*c*_ values are smaller than those averaged for a variety of many 1:1 inclusion complexes built up of αCD host molecules [36]. Nevertheless, an association of second αCD molecule to 1:1 fenchone–αCD complexes significantly increases their stability.

**Table 2:** The association constants for (+)-fenchone–αCD and (−)-fenchone–αCD complexes expressed on the molar (*K*_*c*_) scale

Methyl	*K*_*1,c*_ (M^−1^)	*K*_*2,c*_ (M^−1^)	Δ*δ*_1_ (ppm)	Δ*δ*_2_ (ppm)	F_calc_
(+)-fenchone (*a* ≥ 42.6 ± 3.0)				F_tabl_(2,4;0.01) = 18.0	
C8	9.8 ± 1.6	116.1 ± 17.5	0.897	3.643	478.3
C9	10.0 ± 0.5	104.9 ± 5.9	1.349	2.569	2163
C10	10.0 ± 1.6	109.6 ± 18.6	0.794	1.498	245.0
<C>	10.0 ± 0.5	106.4 ± 5.4			
(−)-fenchone (*a* ≥ 9.9 ± 0.7)				F_tabl_(2,5;0.01) = 13.3	
C8	10.5 ± 1.4	23.2 ± 2.7	0.821	3.437	304.7
C9	7.1 ± 0.9	30.0 ± 5.4	1.937	2.485	98.4
C10	10.5 ± 0.6	23.0 ± 1.4	0.660	1.595	278.1
<C>	9.5 ± 0.5	23.4 ± 1.2			

The complexation ^13^C chemical shift displacements Δ*δ*_1_ and Δ*δ*_2_ for the 1:1 and 1:2 species, respectively, are given along with association constants. <C> denotes the weighted mean calculated from C8–C10 data. The column with the heading F_calc_ indicates the Fisher–Snedecor statistics calculated for a comparison of 1:1 with the sequential 1:1 and 1:2 model. F_calc_ values greater than the corresponding F_tabl_ estimates confirm a meaningful improvement due to an increase in the binding complexity. The cooperativity factor, *a*, has been evaluated from the macroscopic association constants *K*_1,*c*_ and *K*_2,*c*_ (e.g., *a* = 4 *K*_2,*c*_/*K*_1,*c*_) [37]

Association constants expressed on the molar concentration scale, *K*_*i,c*_, are not suitable for determining thermodynamic quantities. Therefore, recalculation of association constants *K*_*i,c*_ on molar fraction scale, *K*_*i,a*_, has to be done [16, 26]. The *K*_*i,a*_ values and estimates of the corresponding Gibbs free energies Δ*G*_0_ for complex formation of both fenchone enantiomers with αCD are given in Table 3. A comparison of these data with earlier results obtained for camphor complexes with αCD [8, 16] reveals that the overall association constants, *β*_12,*a*_ = *K*_1,*a*_·*K*_2,*a*_, for camphor complexes are three orders of magnitude higher than the corresponding values for fenchone complexes. On the other hand, chiral recognition, (i.e., differentiation of enantiomeric species, forming diastereomeric complexes which are, quantitatively expressed as ΔΔ*G*_0_ = Δ*G*_0(−)_ − Δ*G*_0(+)_) for camphor complexes is lower than that observed for fenchone complexes.

**Table 3:** Values of the association constants, (*K*_*i,a*_ *i* = 1,2), in mole fraction scale, Gibbs free energies, Δ*G*_0_, for complex formation of both fenchone enantiomers with αCD and chiral recognition, ΔΔ*G*_0_, compared with corresponding data for camphor complexes taken from Ref. [16]

Enantiomer	*K*_1,*a*_	*K*_2,*a*_	*β*_12,*a*_	Δ*G*_0_ (kJ/mol)	ΔΔ*G*_0_ (kJ/mol)
(+)-fenchone	550.8 ± 26.5	5870 ± 295	(3.23 ± 0.22) 10^6^	−37.5 ± 0.2	4.0 ± 0.2
(−)-fenchone	523.5 ± 26.0	1290 ± 67	(0.68 ± 0.05) 10^6^	−33.5 ± 0.2	
(+)-camphor			(2.07 ± 0.01)·10^9^	−53.6 ± 0.2	2.2 ± 0.2
(−)-camphor			(0.86 ± 0.01)·10^9^	−51.4 ± 0.1	

The overall association constant *β*_*12,a*_ = *K*_*1,a*_ *·K*_*2,a*_

The systems with at least two binding sites can exhibit a complex behavior that depends not only on the affinities for each site but also on the interaction between the sites. For instance, the facing rims of two cyclodextrin molecules may interact forming dimers via hydrogen bonds linking their hydroxyls at C2 and C3 glucopyranose units and promoting additional 1:2 complex stabilization. If the binding to one site enhances the affinity for a second site, the so called positive cooperativity takes place. Since cooperativity factors are specific for microscopic description of multisite association processes, it is not always possible to extract them from macroscopic association constants which are usually obtained experimentally [26, 35, 37]. A qualitative analysis, however, can be performed easily once the macroscopic association constants have been determined. For a system with two binding sites, the cooperativity factor *a* can be estimated from [37]:

$$ a = 4K_{2,c} /K_{1,c} $$

If *a* > 1, the binding sites exhibit positive cooperativity reflecting the favorable energy loss due to a simultaneous host binding to both sites of the guest molecule [26, 37]. One has to bear in mind that this equation is strictly valid only if the two lower order microscopic association constants, *κ*_*1i*_, are identical. It is a consequence of the relation between microscopic and macroscopic association constants: *K*_*1,c*_ = *κ*_*1A*_ + *κ*_*1B*_ [26]. Fortunately, the cooperativity factor reaches a minimum at *κ*_*1A*_ = *κ*_*1B*_ = *K*_*1,c*_/2, where the conclusion about positive cooperativity based on the inequality *a* > 1 remains valid. Therefore, formation of the two chiral (+)- and (−)- fenchone–αCD complexes is characterized by strong cooperativity since their lower limit cooperativity factors are equal to 42.6 and 9.9 for (+)-fenchone and (−)-fenchone complexes, respectively (cf. Table 2). Moreover, it might seem intuitively obvious that a stronger complex is characterized by a larger cooperativity.

The stepwise association constants *K*_*i,c*_ in complexes of fenchone with α-cyclodextrin differ by one order of magnitude. This result is in contrast with the data obtained for corresponding complexes of camphor studied by similar approach [8]. It has been estimated that their stepwise association constants differ by four orders of magnitude, thus, precluding their separation but supporting conclusion about strong cooperative binding in camphor—αCD complexes.

For a qualitative interpretation of complexation ^13^C chemical shifts displacements Δ*δ*_1_ and Δ*δ*_2_, corresponding to the 1:1 and 1:2 complexes, respectively, one should take into account the differential contribution of conformational freedom of guest molecules within the cavity built up of two CD molecules. This may be anticipated from the results obtained for camphor—αCD complexes using NMR relaxation and X-ray studies [10, 17]. Crystallographic studies revealed three distinct guest orientations within host dimer capsule, whereas accompanying MD simulations pointed out to additional camphor fluctuations about its equilibrium orientations within the cavity [17]. Nuclear magnetic relaxation studies confirm fast reorientation of the guest molecules within the αCD capsule in addition to differential intramolecular rotations of the methyl groups [10].

All three methyl carbons in either (+)- or (−)- fenchone–αCD complexes exhibit complexation ^13^C chemical shift displacement for the 1:2 complex (Δ*δ*_2_) that is larger than that of the 1:1 complex (Δ*δ*_1_). One can argue that two αCD molecules surrounding a fenchone molecule may exert stronger perturbation to the environment of a guest molecule than a single αCD molecule, thus resulting in a relatively larger complexation ^13^C chemical shift displacements. In the absence of detailed information on the geometries of fenchone–αCD complexes, however, a detailed interpretation of Δ*δ*_*i*_ values seems problematic. Nevertheless, all but one Δ*δ*_*i*_ values are larger for the more stable (+)-fenchone–αCD complex than for the (−)-fenchone–αCD complex, thus the tighter the complex, the larger is the perturbation and hence the chemical shift displacement.

## Conclusions

Stoichiometry and sequential association constants have been determined for diastereomeric complexes of fenchone enantiomers with α-cyclodextrin by means of NMR titrations. Estimation of stepwise association constants makes it possible to evaluate and confirm the presence of positive cooperativity for 1:2 complex formation, if any.

For both terpenoids, fenchone and camphor, the (+)-enantiomers form more stable complexes with αCD than the corresponding (−)-isomers. Both fenchone complexes, however, are comparatively much less stable than those of camphor. In contrast, chiral recognition by αCD for fenchone is larger in comparison with camphor. It can be expected that the two geminal methyl groups attached to the C3 carbon atom in fenchone impose more steric hindrance to complex formation with αCD than their counterparts in camphor located at the C7 carbon.

## Electronic supplementary material

Below is the link to the electronic supplementary material.

Supplementary material 1 (JPEG 455 kb)

Supplementary material 2 (JPEG 456 kb)

Supplementary material 3 (JPEG 449 kb)

Supplementary material 4 (JPEG 894 kb)

## References

[1] Del Valle EMM (2004). Cyclodextrins and their uses: a review. Proc. Biochem..

[2] Szejtli J (1998). Introduction and general overview of cyclodextrin chemistry. Chem. Rev..

[3] Loftsson T, Duchene D (2007). Cyclodextrins and their pharmaceutical applications. Int. J. Pharm..

[4] Liu L, Guo QX (2002). The driving forces in the inclusion complexation of cyclodextrins. J. Incl. Phenom. Macrocycl. Chem..

[5] Ejchart A, Koźmiński W, Dodziuk H (2006). NMR of cyclodextrins and their complexes. Cyclodextrins and their Complexes.

[6] Bielejewska A, Nowakowski R, Duszczyk K, Sybilska D (1999). Joint use of cyclodextrin additives in chiral discrimination by reversed-phase high-performance liquid chromatography: temperature effects. J. Chromatogr..

[7] Asztemborska M, Bielejewska A, Duszczyk K, Sybilska D (2000). Comparative study on camphor enantiomers behavior under the conditions of gas-liquid chromatography and reversed-phase high performance liquid chromatography systems modified with alpha and beta-cyclodextrins. J. Chromatogr. A.

[8] Dodziuk H, Ejchart A, Lukin O, Vysotsky MO (1999). ^1^H and ^13^C NMR and molecular dynamics study of chiral recognition of camphor enantiomers by α-cyclodextrin. J. Org. Chem..

[9] Schmidtchen FP (2002). The anatomy of the energetics of molecular recognition by calorimetry: chiral discrimination of camphor by α-cyclodextrin. Chem. Eur. J..

[10] Anczewski W, Dodziuk H, Ejchart A (2003). Manifestation of chiral recognition of camphor enantiomers by α-cyclodextrin in longitudinal and transverse relaxation rates of the corresponding 1:2 complexes and determination of the orientation of the guest inside the host capsule. Chirality.

[11] Dodziuk H, Koźmiński W, Dolgonos G (2003). The differences between the Δ*H* and Δ*S* values of the 1:2 complex of camphor enantiomers with α-cyclodextrin determined by NMR titration and the results obtained by other techniques. Pol. J. Chem..

[12] Dodziuk H, Nowiński KS, Koźmiński W, Dolgonos G (2003). On the impossibility of determination of stepwise binding constants for the 1:2 complex of (+)-camphor with α-cyclodextrin. Org. Biomol. Chem..

[13] Simova S, Berger S (2005). Diffusion measurements versus chemical shift titration for determination of association constants on the example of camphor-cyclodextrin complexes. J. Incl. Phenom. Macrocycl. Chem..

[14] Liu Y, Yang E-C, Yang Y-W, Zhang H-Y, Fan Z, Ding F, Cao R (2004). Thermodynamics of the molecular and chiral recognition of cycloalkanols and camphor by modified β-cyclodextrins possessing simple aromatic tethers. J. Org. Chem..

[15] Liu Y, Zhang Q, Chen Y (2007). Spectrophotometric and calorimetric titration studies on molecular recognition of camphor and borneol by nucleobase-modified β-cyclodextrins. J. Phys. Chem. B.

[16] Bernatowicz P, Nowakowski M, Dodziuk H, Ejchart A (2006). Determination of association constants at moderately fast chemical exchange: complexation of camphor enantiomers by α-cyclodextrin. J. Magn. Reson..

[17] Kokkinou A, Tsorteki F, Karpusas M, Papakyriakou A, Bethanis K, Mentzafos D (2010). Study of the inclusion of the (R)- and (S)-camphor enantiomers in α-cyclodextrin by X-ray crystallography and molecular dynamics. Carbohydr. Res..

[18] Donze C, Coleman AW (1993). β-CD inclusion complexes: relative selectivity of terpene and aromatic guest molecules studied by competitive inclusion experiments. J. Incl. Phenom. Macrocycl. Chem..

[19] Sybilska D, Asztemborska M (2002). Chiral recognition of terpenoids in some pharmaceuticals derived from natural sources. J. Biochem. Biophys. Methods.

[20] Kikuchi T, Dorjpalam N, Endo K, Hamada F (2003). Synthesis and guest binding properties of regioselectivity athranilate-tosyl-labeled β-cyclodextrins. Int. J. Soc. Mater. Eng. Resour..

[21] Skórka M, Asztemborska M, Zukowski J (2005). Thermodynamic studies of complexation and enantiorecognition processes of monoterpenoids by alpha- and beta-cyclodextrin in gas chromatography. J. Chromatogr. A.

[22] Zalachoras I, Kagiava A, Vokou D, Theophilidis G (2010). Assessing the local anesthetic effects of five essential oil constituents. Planta Med..

[23] Vummaneni V, Nagpal D (2012). Taste masking technologies: an overview and recent updates. Int. J. Res. Pharm. Biomed. Sci..

[24] Fielding L (2000). Determination of association constants (*K*_*a*_) from solution NMR data. Tetrahedron.

[25] Hirose K (2001). A practical guide for the determination of binding constants. J. Incl. Phenom. Macrocycl. Chem..

[26] Connors KA (1997). The stability of cyclodextrin complexes in solution. Chem. Rev..

[27] Schneider HJ, Hacket F, Rüdiger V, Ikeda H (1998). NMR studies of cyclodextrins and cyclodextrin complexes. Chem. Rev..

[28] Feeney J, Batchelor JG, Albrand JP, Roberts GCK (1979). The effect of intermediate exchange processes on the estimation of equilibrium constants by NMR. J. Magn. Reson..

[29] Dodziuk H, Koźmiński W, Ejchart A (2004). NMR studies of chiral recognition by cyclodextrins. Chirality.

[30] Gottlieb HE, Kotlyar V, Nudelman A (1997). NMR chemical shifts of common laboratory solvents as trace impurities. J. Org. Chem..

[31] Press WH, Flannery BP, Teukolsky SA, Vetterling WT (1986). Numerical Recipes. The Art of Scientific Computing.

[32] Kolehmainen E, Laihia K, Korvola J, Kauppinen R, Pitkanen M, Mannila B, Mannila E (1990). Multinuclear NMR study of 1,3,3-trimethylbicyclo[2.2.1] heptan-2-one (fenchone) and its six monochlorinated derivatives. Magn. Reson. Chem..

[33] Abraham, R.J., Ainger, N.J.: Proton chemical shifts in NMR. Part 13. Proton chemical shifts in ketones and the magnetic anisotropy and electric field effect of the carbonyl group. *J. Chem. Soc.*, *Perkin Trans.**2*, 441–448 (1999)

[34] Wyman J, Phillipson P (1974). A probabilistic approach to cooperativity of ligand binding by a polyvalent molecule. Proc. Natl. Acad. Sci. USA.

[35] Holt JM, Ackers GK (2009). The Hill coefficient: inadequate resolution of cooperativity in human hemoglobin. Methods Enzymol..

[36] Connors KA (1995). Population characteristics of cyclodextrin complex stabilities in aqueous solution. J. Pharm. Sci..

[37] Freire E, Schön A, Velazquez-Campoy A (2009). Isothermal titration calorimetry: general formalism using binding polynomials. Methods Enzymol..

