# Supplementary figures and images for: Complex formation of fenchone with α-cyclodextrin: NMR titrations
Source: J Incl Phenom Macrocycl Chem. 2013 Aug 10;79(3):337–42. doi: 10.1007/s10847-013-0356-4 (PMC4082655; doi:10.1007/s10847-013-0356-4)

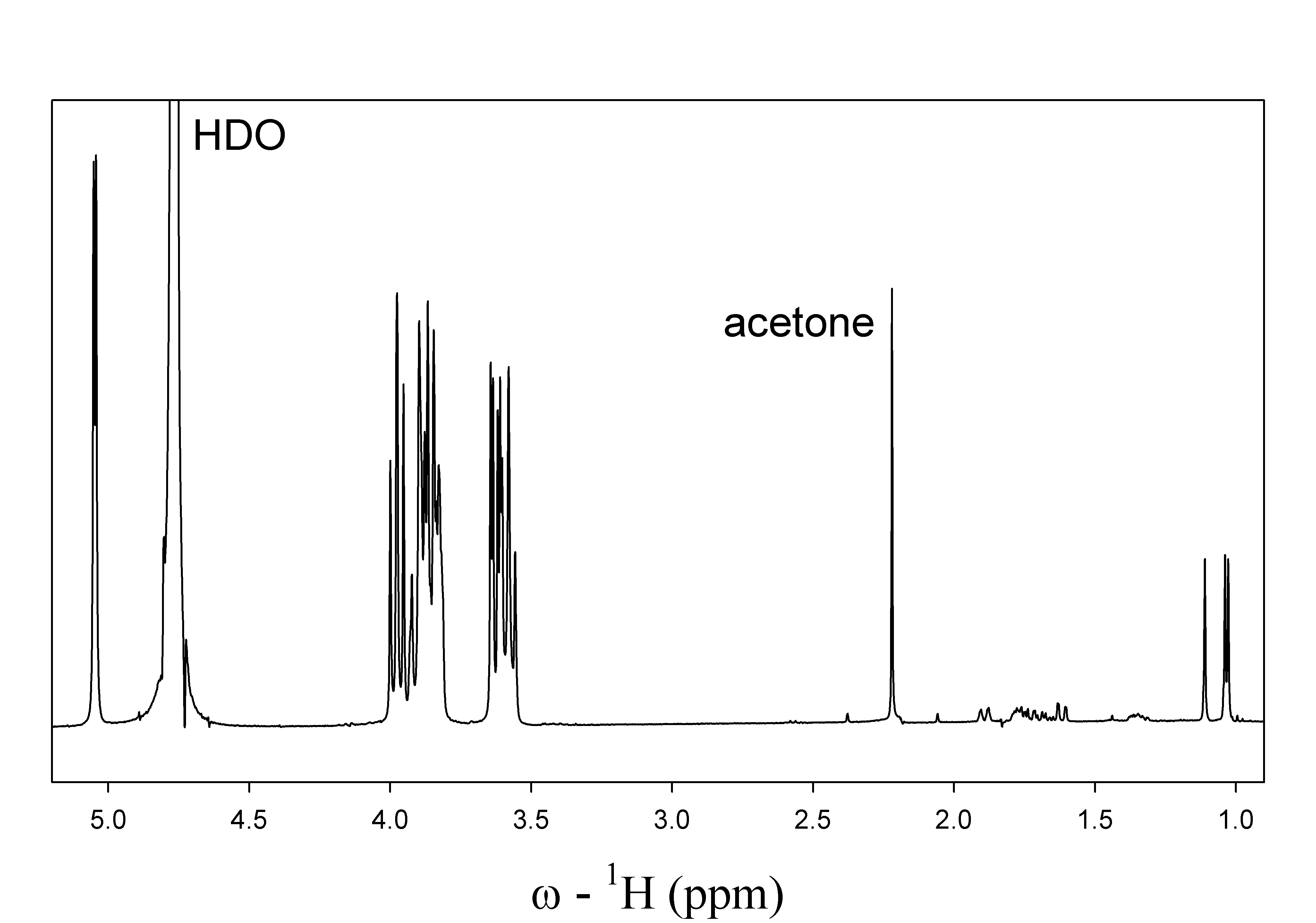

Supplement: Supplementary file 1 — Supplementary material 1 (JPEG 455 kb) [file 10847_2013_356_MOESM1_ESM.jpg]

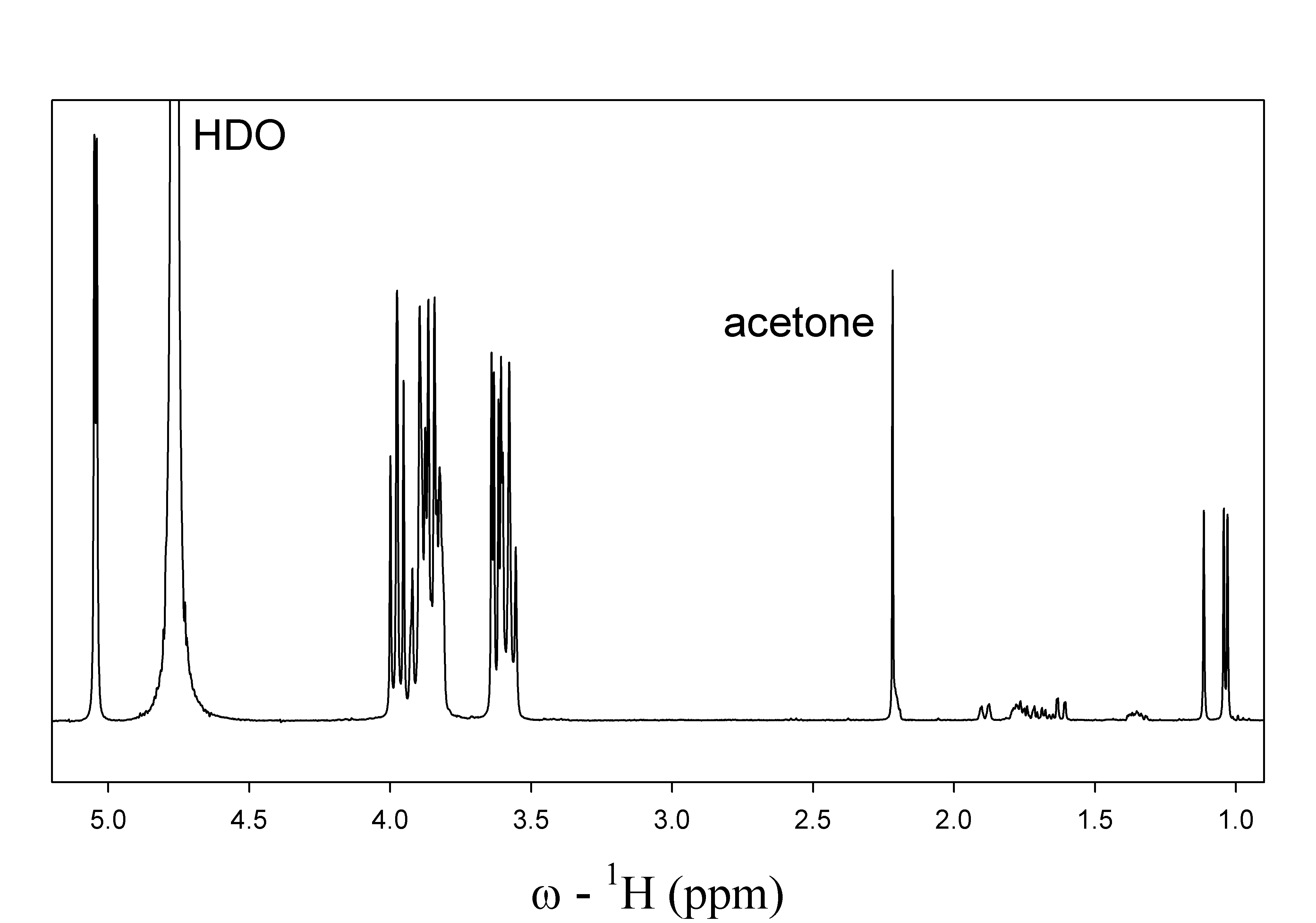

Supplement: Supplementary file 2 — Supplementary material 2 (JPEG 456 kb) [file 10847_2013_356_MOESM2_ESM.jpg]

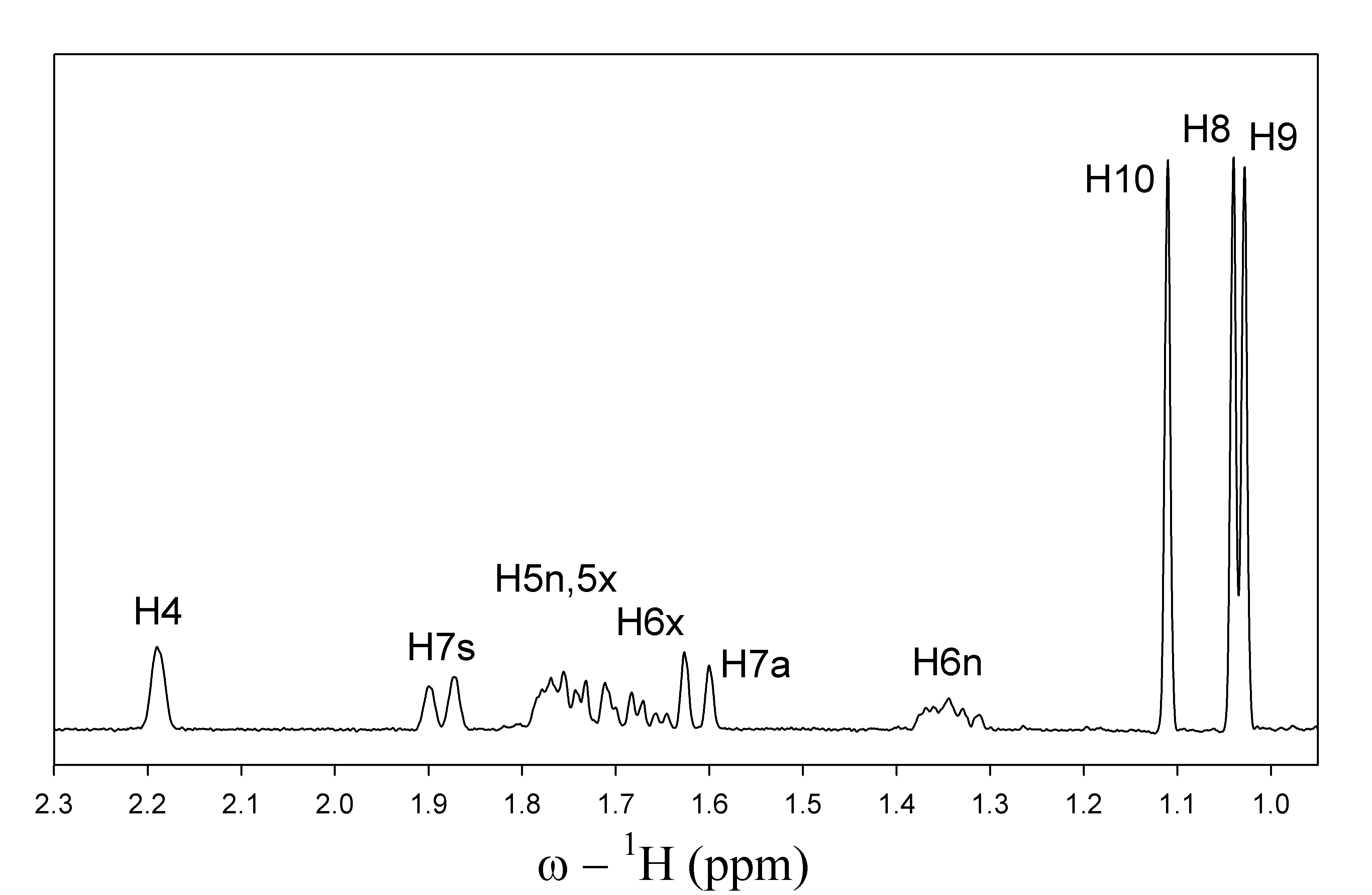

Supplement: Supplementary file 3 — Supplementary material 3 (JPEG 449 kb) [file 10847_2013_356_MOESM3_ESM.jpg]
